# Development and validation of the Chinese version of dry eye related quality of life scale

**DOI:** 10.1186/s12955-017-0718-5

**Published:** 2017-07-17

**Authors:** Bang Zheng, Xiao-jing Liu, Yue-qian Fiona Sun, Jia-zeng Su, Yang Zhao, Zheng Xie, Guang-yan Yu

**Affiliations:** 1 Peking University School of Public Health, Beijing, 100191 China; 2 Department of Oral and Maxillofacial Surgery, Peking University School & Hospital of Stomatology, Beijing, 100081 China; 3 London School of Hygiene & Tropical Medicine, London, WC1E 7HT UK; 4 Beijing Tongren Eye Center, Beijing Key Laboratory of Ophthalmology and Visual Science, Beijing Tongren Hospital, Capital Medical University, Beijing, 100730 China

**Keywords:** Dry eye syndrome, Health-related quality of life, Scale, Reliability, Validity

## Abstract

**Background:**

To develop the Chinese version of quality of life scale for dry eye patients based on the Impact of Dry Eye on Everyday Life (IDEEL) questionnaire and to assess the reliability and validity of the developed scale.

**Methods:**

The original IDEEL was adapted cross-culturally to Chinese language and further developed following standard procedures. A total of 100 Chinese patients diagnosed with dry eye syndrome were included to investigate the psychometric properties of the Chinese version of scale. Psychometric tests included internal consistency (Cronbach’s ɑ coefficients), construct validity (exploratory factor analysis), and known-groups validity (the analysis of variance).

**Results:**

The Chinese version of Dry Eye Related Quality of Life (CDERQOL) Scale contains 45 items classified into 5 domains. Good to excellent internal consistency reliability was demonstrated for all 5 domains (Cronbach’s ɑ coefficients range from 0.716 to 0.913). Construct validity assessment indicated a consistent factorial structure of the CDERQOL scale with hypothesized construct, with the exception of “Dry Eye Symptom-Bother” domain. All domain scores were detected with significant difference across three severity groups of dry eye patients (*P* < 0.05) except for “Satisfaction with Treatment” domain, indicating good known-groups validity.

**Conclusions:**

The results indicated that the CDERQOL scale is a reliable and valid instrument for patients with dry eye syndrome among Chinese population, and could be used as a supplementary diagnostic and treatment-effectiveness measure.

**Electronic supplementary material:**

The online version of this article (doi:10.1186/s12955-017-0718-5) contains supplementary material, which is available to authorized users.

## Background

Dry eye syndrome is a relatively common disease of the tear film and ocular surface that results in eye discomfort, visual disturbance, and often ocular surface damage [1, 2]. Previous researches showed dry eye syndrome has long been prevalent in Chinese population [3, 4]. A recently published meta-analysis showed the pooled prevalence of dry eye syndrome in mainland of China was 17.0%, among which female and elder individuals were more likely to suffer [5]. The current therapeutic options are largely symptomatic, including artificial tear substitutes, or occlusion of the tear drainage. These treatment modalities give satisfactory results in mild cases. However, they seem to be insufficient in severe cases. Severe dry eye could cause corneal ulceration, opacification, even blindness. Microvascular autologous transplantation of the submandibular gland (SMG) has been suggested to be an effective treatment for severe cases of dry eye syndrome [6–9].

Due to its symptom-based nature, dry eye syndrome could have detrimental effects on patients’ health-related quality of life (HRQL) [10]. In this regard, it seems to be necessary that the diagnosis and evaluation of the severity of the disease depend not only on objective clinical tests, but also assessments of perceived symptoms, as well as patients’ quality of life [11, 12]. There has been only a few well-developed quality of life scales specific on dry eye patients [13], including the Ocular Surface Disease Index (OSDI) [14], the Impact of Dry Eye on Everyday Life (IDEEL) questionnaire [15], and a newly developed single-item questionnaire named the University of North Carolina Dry Eye Management Scale (UNC DEMS) [16]. Among these dry eye scales, the IDEEL is considered to be a reliable and validated questionnaire that fully assesses symptoms together with the effect of dry eye on daily life. The IDEEL generated in American English contains 57 items organized into 3 modules, covering 6 relevant domains which are Dry Eye Symptom Bother, Dry Eye Impact on Daily Life (impact on daily activities, emotional impact due to dry eye, impact on work due to dry eye), and Dry Eye Treatment Satisfaction (satisfaction with treatment effectiveness and treatment-related bother/inconvenience) [15].

Nevertheless, no dry eye-specific quality of life scale has been developed in China yet that aims to assess the severity of dry eye syndrome and to evaluate the clinical effects of surgical treatment for severe dry eye patients. Therefore, the purpose of this study was to develop a Chinese version of quality of life scale for dry eye patients based on the IDEEL questionnaire and to assess the reliability and validity of the developed scale.

## Methods

### Study population

Participants were recruited between December 2013 and July 2015 in two specialist hospitals in Beijing, China. Patients’ dry eye status was evaluated by an ophthalmologist using the Schirmer test, break-up time, rose bengal staining and fluorescence staining [6]. After confirmed diagnosis of dry eye syndrome, patients were asked to complete a self-administered questionnaire to test the pilot version of the dry eye scale under the direction of a trained research assistant. For those who did not have enough time or those who had vision defect, telephone interviews were conducted instead during the routine follow-up check by the attending physicians.

Inclusion criteria were: adult subjects ≥18 years, participation on voluntary basis, literate in Chinese. Patients were excluded from the study if they were diagnosed with Sjögren syndrome, or if they had other severe diseases that may influence their quality of life substantially, such as cancer or stroke.

### Ethical approval

The study was approved by the Institutional Review Board of the Peking University Health Science Center (No. IRB00001052–08048). All of the participants signed an informed consent form prior to the study.

### Translation and development of the scale

**Fig. 1:**
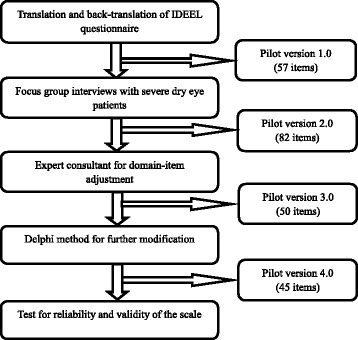
The developing procedure of the CDERQOL scale

The procedure of scale development was shown in Fig. 1. A standard forward-backward procedure was used for the translation of original questionnaire. First, two forward translators independently translated the IDEEL questionnaire into Chinese (initial Version 1.0 with 57 items), and then two backward translators translated it back into English in order to maintain accuracy between the two languages and achieve equivalence between resource and target questionnaires [17]. The researchers contacted a professional translation agency to choose those four translators. All of the translators were native speakers of Chinese who are fluent in English and also familiar with medical terminology. The researchers compared two translation and discussed with the translators for consensus.

In order to achieve cross-culture adaptation [17], we further conducted three focus group interviews with six patients with severe dry eye syndrome in each group. A topic guide was used for the semi-structured interviews, which included three main questions: (1) what are the symptoms of dry eye bothering you? (2) what are the impact of dry eye on your daily life? (3) Do you feel satisfied with the treatment and why? Interviews were transcribed into text and data were coded and analyzed by two experienced qualitative researchers of the team using thematic framework approach [18]. A thematic framework was used for coding (Table 1). New emerging themes from the interviews were added in order to achieve completeness and comprehension of the questionnaire for next stage work. Those items not relevant in Chinese context were deleted. Eventually, we got an 82-item Version 2.0 scale.

**Table 1:** Coding thematic framework for interviews

1. Physical discomfort caused by dry eye	
2. Impact of dry eye on daily life	
2.1 impact on daily activities	
2.2 emotional impact	
2.3 impact on work	
3. Satisfaction with treatment	
3.1 satisfaction with the result of treatment	
3.2 Inconvenience of treatment	

Later two ophthalmologists, three stomatologists and one public health expert were invited to further adjust the scale domains and revise the items. As a result, 30 items were deleted, 5 items were combined, 16 items were revised in wording and 3 items were moved to other domains, which turned into scale Version 3.0.

Lastly, Delphi inquiry method was used to collect both qualitative and quantitative assessments from 30 experts of ophthalmology (10), stomatology (10) and public health (10). We asked experts to rate each item from Version 3 a score between 1 to 5 according to its comprehensiveness and clarity. We also invited them to give suggestions for further modification. Finally, several additional deletions and additions were made, yielding pilot scale Version 4.0, which was used to assess scale’s reliability and validity.

The pilot Chinese version of Dry Eye Related Quality of Life (CDERQOL) Scale contains 45 items classified into 5 domains: Dry Eye Symptom Bother (12 items), Dry Eye Impact on Daily Life (impact on daily activities, 7 items; emotional impact, 10 items; impact on work, 7 items), and Satisfaction with Treatment (9 items). Each item was measured using a 5-point Likert scale ranging from “completely disagree” (1) to “completely agree” (5) [19, 20].

### Statistical analysis

We first described the sex and age distributions of study population, and examined differences of sex and age-group proportions across three dry-eye severity groups using chi-square tests. Then we evaluated the reliability and validity of the scale using multiple psychometric properties and tests. In detail, the construct validity was evaluated using exploratory factor analysis (EFA), which combined principal components analysis (PCA) with promax rotation analysis to testify the conceptual framework and item-dimension structures of the scale. In order to test whether the data was suitable for factor analysis, the Kaiser-Meyer-Olkin Measure of Sampling Adequacy test and the Bartlett test of sphericity were conducted before we performed EFA. Factors were retained if their eigenvalues were above 1.0 or according to the scree plot, and in combination with the theoretical structure of the scale. We considered item loadings satisfactory if loadings were above 0.40 with their own factor and larger than loadings with other factors [21]. The internal consistency reliability was assessed using Cronbach’s ɑ coefficients. The analysis of variance (ANOVA) was then conducted to evaluate the known-groups validity, which means to test for differences in domain scores among patients with different levels of dry eye severity. We also performed post-hoc pairwise comparisons using Least Significant Difference tests.

Statistical analysis was performed using PASW Statistics 18 (SPSS, Chicago, IL). Where applicable, a *P* value of less than 0.05 was considered statistically significant.

## Results

### Study population demographics

The initial sample size was 100. After exclusion of missing data or abnormal value, 90 subjects remained in the analyses. The demographic characteristics of the study participants are presented in Table 2.

**Table 2:** Demographic characteristics of the study population (*N* = 90)

Characteristics		Severity of dry eye patients, *n* (%)^a^			*χ* ^2^ value	*P* value
		Mild (*n* = 24)	Moderate (*n* = 36)	Severe (*n* = 30)		
Age	< 35	2 (8.3)	10 (27.8)	16 (66.7)	21.915	< 0.001
	[35,55)	8 (33.3)	9 (25.0)	6 (25.0)		
	≥ 55	14 (58.3)	17 (47.2)	2 (8.3)		
Sex	Male	7 (29.2)	10 (27.8)	15 (53.6)	5.267	0.072
	Female	17 (70.8)	26 (72.2)	13 (46.4)		

^a^Some variables contained missing values, thus the proportion in bracket () was valid proportion; severity groups were classified according to clinical diagnosis

Among the 90 participants, 60 were diagnosed with “mild or moderate dry eye syndrome”, while 30 with “severe dry eye syndrome”. Among the 30 severe patients, the etiologies were Stevens–Johnson syndrome in 25 patients, acute conjunctivitis in 3 patients, corneal pemphigoid in 1 patient, corneal chemical burns in 1 patient, and unknown in 4 patients.

The majority of the population was female (71.7%) and the mean age was 50.0 ± 13.8 years (range: 20–70 years old). Results of chi-square tests showed that the “severe dry eye” group was significantly younger than both “mild dry eye” and “moderate dry eye” groups, while the “mild” and “moderate groups” did not differ significantly by age. As for sex, the “severe group” contained a significantly higher proportion of male subjects (53.6%) than the other two groups, while the “mild” and “moderate groups” did not differ significantly by sex.

### Psychometric validation of the scale

#### Construct validity

Given that three modules of “Dry Eye Symptom Bother”, “Dry Eye Impact on Daily Life” and “Satisfaction with Treatment” belong to different concepts [22, 23], it was hypothesized that each module could develop its own set of items and be considered as distinct sub-scales [15]. Therefore, we conducted factor analysis of each module separately.

The three domains of Impact on Daily Activities, Emotional Impact and Impact on Work set up the “Dry Eye Impact on Daily Life” module. Results of the Kaiser-Meyer-Olkin Measure of Sampling Adequacy test and the Bartlett test of sphericity indicated that the data was suitable for factor analysis (KMO = 0.840; Bartlett = 1527.1, *P* < 0.001). We then conducted the factor analysis using PCA method and obtained 5 eigenvalues >1. But according to the scree plot, it was reasonable to extract 3 common factors, of which the accumulated variance contribution rate was up to 59.6% (Table 3).

**Table 3:** Eigenvalues of the common factors and total variance explained

Components	Extraction sums of squared loading			Rotation sums of squared loading		
	Eigenvalue	Variance contribution rate, %	Accumulated variance contribution rate, %	Eigenvalue	Variance contribution rate, %	Accumulated variance contribution rate, %
1	10.34	43.07	43.07	5.09	21.22	21.22
2	2.33	9.72	52.79	4.82	20.08	41.30
3	1.64	6.82	59.61	4.40	18.32	59.61
4	1.33	5.55	65.16			
5	1.06	4.41	69.58			
6	0.96	3.99	73.56			
7	0.80	3.35	76.91			
8	0.75	3.11	80.02			
9	0.66	2.76	82.78			
10	0.60	2.50	85.28			
11	0.53	2.22	87.50			
12	0.47	1.95	89.45			
13	0.42	1.74	91.19			
14	0.35	1.46	92.65			
15	0.32	1.34	93.99			
16	0.27	1.14	95.13			
17	0.23	0.95	96.08			
18	0.22	0.93	97.00			
19	0.19	0.78	97.78			
20	0.17	0.71	98.50			
21	0.12	0.52	99.01			
22	0.11	0.47	99.48			
23	0.08	0.34	99.82			
24	0.04	0.18	100.00			

We further conducted promax rotation analysis to distinguish the 3 common factors. The results showed that most items of Emotional Impact could be explained by factor 1, all items of Impact on Work could be explained by factor 2, and most items of Impact on Daily Activities could be explained by factor 3, which suggested that the construct validity for the “Dry Eye Impact on Daily Life” module was good (Table 4). Although several items didn’t load well on their own factors or loaded high on more than one factor, we didn’t make further adjustments to items so as to retain comparability with the original scale.

**Table 4:** Rotated factor matrix of the Dry Eye Impact on Daily Life module (promax method)

Domains	Items	Factors		
		1	2	3
Impact on Daily Activities	B1	0.363	**0.401**	0.139
	B2	0.217	−0.157	**0.686**
	B3	0.225	0.440	**0.471**
	B4	0.244	0.288	**0.758**
	B5	−0.024	0.148	**0.756**
	B6	0.261	0.367	**0.405**
	B7	0.389	0.286	**0.600**
Emotional Impact	C1	**0.659**	0.243	0.284
	C2	**0.734**	0.346	0.199
	C3	**0.624**	0.316	0.273
	C4	**0.805**	0.039	0.463
	C5	0.389	**0.532**	−0.008
	C6	0.369	**0.441**	0.440
	C7	**0.739**	0.059	0.546
	C8	**0.701**	0.310	0.079
	C9	**0.789**	0.220	0.185
	C10	0.449	0.074	**0.632**
Impact on Work	D1	0.191	**0.782**	0.232
	D2	0.020	**0.841**	0.114
	D3	0.214	**0.766**	0.243
	D4	0.370	**0.462**	0.154
	D5	0.301	**0.586**	0.497
	D6	0.286	**0.579**	−0.273
	D7	0.179	**0.640**	0.448

Note: values in boldface indicate the highest factor loading of the item

For “Dry Eye Symptom Bother” module, the results suggested 2 dimensions, which was not quite consistent with the theoretical conceptual framework. The accumulated variance contribution rate of the 2 factors was only 45.0%. The results are presented in (Additional file 1: Tables S1 and S2).

The “Satisfaction with Treatment” module yielded 2 distinct factors with eigenvalues >1, of which the accumulated variance contribution rate was up to 60.1%, indicating a structure of 2 dimensions. The dimensions could be clinically interpreted as Satisfaction with Treatment Effectiveness and Satisfaction with Tear Amount. The results are presented in (Additional file 1: Tables S3 and S4).

#### Internal consistency reliability

The internal consistency reliability of the scale is presented in Table 5. The internal consistency reliability was acceptable in all 5 domains (Dry Eye Symptom Bother, Impact on Daily Activities, Emotional Impact, Impact on Work and Satisfaction with Treatment), with the Cronbach’s ɑ coefficients ranging from 0.716 to 0.913.

**Table 5:** Internal consistency properties (Cronbach’s ɑ coefficients) of the CDERQOL scale

Domains	Number of items	Cronbach’s ɑ coefficients
Dry Eye Symptom Bother	12	0.826
Impact on Daily Activities	7	0.811
Emotional Impact	10	0.913
Impact on Work	7	0.865
Satisfaction with Treatment	9	0.716

#### Known-groups validity

The mean scores of all domains were found significantly different across three dry-eye severity levels (*P* < 0.001), except for the domain of Satisfaction with Treatment (Table 6). Results of pairwise comparisons showed that for domains of Dry Eye Symptom Bother and Impact on Daily Activities, the scores of mild and moderate patients group both differed significantly from that of the severe group (*P* < 0.05). As for domains of Emotional Impact and Impact on Work, all pairwise comparisons between the three severity groups resulted in *P* < 0.05. The results suggested the known-groups validity of the CDERQOL scale was good.

**Table 6:** Known-groups validity of the CDERQOL scale by dry eye severity

Domain scores $\overline{x}$ (*s*)	Severity of dry eye^*^			*F* value	*P* value
	Mild (*n* = 24)	Moderate (*n* = 36)	Severe (*n* = 30)		
Dry Eye Symptom Bother	32.7 (8.4)	35.3 (9.8)	44.1 (9.0)^b,c^	12.028	< 0.001
Impact on Daily Activities	17.4 (4.6)	19.8 (5.1)	28.8 (3.9)^b,c^	47.476	< 0.001
Emotional Impact	21.2 (7.5)	25.9 (7.2)^a^	38.4 (7.5)^b,c^	40.864	< 0.001
Impact on Work	20.0 (5.8)	23.3 (6.8)^a^	27.0 (5.9)^b,c^	8.898	< 0.001
Satisfaction with Treatment	31.3 (6.0)	32.5 (4.5)	30.3 (5.1)	1.333	0.270

^*^Severity groups were classified according to clinical diagnosis

^a^ *P* < 0.05 for pairwise comparison between mild and moderate groups

^b^ *P* < 0.05 for pairwise comparison between mild and severe groups

^c^ *P* < 0.05 for pairwise comparison between moderate and severe groups

## Discussion

This study is one of the first studies that targeted at development and validation of the Chinese version of quality of life scale among dry eye patients. We have fulfilled the translation and cross-cultural adaptation of the widely-used IDEEL questionnaire. The results of psychometric analyses showed that the CDERQOL scale obtained good reliability and validity among Chinese dry eye patients.

In contrast to the IDEEL, the CDERQOL scale was developed based on the symptom complaints and life quality situation of Chinese patients, and emphasized more on the severe group of patients. In consideration of the severe patients’ symptoms and feelings, we added specific items such as “Vision loss”, “Little or no tears when crying” and “Lose confidence in treatment”. Moreover, the items “Afraid of economic burden of the treatment for dry eye” and “Afraid of trouble brought to your family members” were added in consideration of social-support for dry eye patients. Since more patients in China are in or over their middle ages than in other countries [5], we deleted the items of “Wearing contact lenses” and “Wearing make-up near or on my eyes” in the original IDEEL according to the Chinese patients’ daily lifestyle and social context. We also replaced the item “Working on a computer” with “Watching TV for a long time” because computers have not yet been widely used in some parts of China, especially in rural areas. When we interviewed Chinese patients and experts, we realized few reported “Headaches associated with dry eye symptoms” and the emotion of “Feeling older than I really are”, so we deleted the items based on the information from focus group interviews with patients and expert consultation.

The reliability analyses performed in this study indicated the CDERQOL scale has high internal consistency within all 5 domains, which is consistent with the original IDEEL. To be noted, the Cronbach’s ɑ coefficients demonstrated relatively low reliability of the domain “Satisfaction with Treatment”, with similar results also found in IDEEL [15]. This may be due to the relatively distinct aspects of items in this domain. However, the coefficients we got all surpassed the empirical reliability criterion of 0.70 [24, 25], which confirmed strong internal reliability of the scale.

As for the validity assessment, the construct validity results of the CDERQOL scale were basically consistent with the hypothesized conception structure except for the “Dry Eye Symptom Bother” domain. The factor analysis results indicated two dimensions for “Dry Eye Symptom Bother”. But the two factors only accounted for 45.0% of the total variance, which means they cannot fully represent or explain the whole set of items, thus need further investigation. Same with the IDEEL, “Satisfaction with Treatment” domain yielded two distinct factors, but we focused more on “Satisfaction with Tear Amount” after the treatment instead of the “Treatment-Related Bother/Inconvenience” in the original IDEEL questionnaire.

The known-groups validity of all domains was good except for the domain of “Satisfaction with Treatment”. This is highly understandable because there are currently limited treatment methods for dry eye syndrome and treatment effects have not been confirmed yet. Thus there could be little difference of the treatment satisfaction among different severity groups of patients.

The current study still has several limitations. First of all, the sample size for psychometric validation was relatively small due to practical difficulties in recruiting eligible dry eye patients in hospitals, which could possibly compromise our results. However, some studies demonstrated that parametric statistics remain robust even with small sample size in the psychometric analysis [26]. Secondly, instead of in-person interview, a few patients in our study were interviewed through telephone due to realistic conditions, which could possibly lead to information bias [27]. There also remains a methodology problem questioning whether it is appropriate to use parametric methods (such as ANOVA and EFA conducted above) to analyze ordinal Likert Scale data. In fact, there is still a lot of discussion on this topic [26, 28]. Moreover, some researches indicated Likert scale could be biased under different cultural background and has so-called “reference-group effect” [29–31], which were not detected in this study. Finally, after the process of cross-cultural adaptation and item modification, the Chinese version of scale may not be reliably comparable with the original version, although most of the items overlap between the two versions.

In light of next steps, further research into the comparability between the Chinese version of dry eye scale and original IDEEL is warranted. Moreover, as the Chinese population is experiencing dramatic change in lifestyle and social context, some items of the scale may not be suitable in the future. Therefore, future research is needed to evaluate the content validity of the scale. Besides of the psychometric properties assessed in this study, several other properties merit further investigation, such as test-retest reliability, concurrent validity and responsiveness. Furthermore, it is worth consideration to combine the CDERQOL scale with clinical tests of dry eye syndrome in clinical research.

## Conclusions

The CDERQOL scale we developed in this study has been proved to be a reliable and valid instrument for the measurement of Quality of Life of Chinese dry eye patients. The scale could have various applications including diagnosis and severity assessment of dry eye syndrome, as well as the evaluation of treatment effectiveness among dry eye patients.

## Additional file

Additional file 1: Results of exploratory factor analysis for “Dry Eye Symptom Bother” and “Satisfaction with Treatment” modules. (DOCX 18 kb)

## Abbreviations

ANOVA: Analysis of Variance
CDERQOL: Chinese version of Dry Eye Related Quality of Life Scale
EFA: Exploratory Factor Analysis
HRQL: Health-related Quality of Life
IDEEL: Impact of Dry Eye on Everyday Life
OSDI: Ocular Surface Disease Index
PCA: Principal Components Analysis
SMG: Submandibular Gland
UNC DEMS: University of North Carolina Dry Eye Management Scale
