# Supplementary material for: Development and validation of the Chinese version of dry eye related quality of life scale
Source: Health Qual Life Outcomes. 2017 Jul 17;15:145. doi: 10.1186/s12955-017-0718-5 (PMC5512764; doi:10.1186/s12955-017-0718-5)
Supplement: Additional file 1: — Results of exploratory factor analysis for “Dry Eye Symptom Bother” and “Satisfaction with Treatment” modules. (DOCX 18 kb) [file 12955_2017_718_MOESM1_ESM.docx]

**Additional Files**

**Additional file 1:** **Results of exploratory factor analysis for “****Dry Eye Symptom Bother” and “Satisfaction with Treatment” modules**

**Supplementary table 1. Eigenvalues of the common factors and total variance explained for “Dry Eye Symptom Bother” module**

| Components | Extraction sums of squared loading | | | Rotation sums of squared loading | | |
| --- | --- | --- | --- | --- | --- | --- |
|  | Eigenvalue | Variance contribution rate, % | Accumulated variance contribution rate, % | Eigenvalue | Variance contribution rate, % | Accumulated variance contribution rate, % |
| 1 | 4.20 | 34.98 | 34.98 | 2.85 | 23.74 | 23.74 |
| 2 | 1.20 | 10.02 | 45.00 | 2.55 | 21.26 | 45.00 |
| 3 | 1.08 | 9.01 | 54.01 |  |  |  |
| 4 | 1.03 | 8.62 | 62.63 |  |  |  |
| 5 | 0.86 | 7.19 | 69.81 |  |  |  |
| 6 | 0.78 | 6.50 | 76.31 |  |  |  |
| 7 | 0.68 | 5.66 | 81.97 |  |  |  |
| 8 | 0.58 | 4.86 | 86.83 |  |  |  |
| 9 | 0.55 | 4.58 | 91.41 |  |  |  |
| 10 | 0.36 | 3.00 | 94.40 |  |  |  |
| 11 | 0.35 | 2.91 | 97.31 |  |  |  |
| 12 | 0.32 | 2.69 | 100.00 |  |  |  |

Note: According to the eigenvalues and the scree plot, two common factors were extracted.

**Supplementary table 2. Rotated factor matrix of the “Dry Eye Symptom Bother” module (promax method)**

| Items | Factors | | |
| --- | --- | --- | --- |
|  | 1 | 2 |  |
| A1 | **0.551** | 0.283 |  |
| A2 | **0.698** | 0.345 |  |
| A3 | **0.575** | 0.289 |  |
| A4 | **0.687** | -0.179 |  |
| A5 | **0.461** | 0.430 |  |
| A6 | **0.513** | 0.055 |  |
| A7 | 0.167 | **0.688** |  |
| A8 | **0.498** | 0.434 |  |
| A9 | 0.302 | **0.663** |  |
| A10 | -0.038 | **0.679** |  |
| A11 | 0.221 | **0.631** |  |
| A12 | **0.602** | 0.295 |  |

Note: values in boldface indicated the highest factor loading of the item.

**Supplementary table 3. Eigenvalues of the common factors and total variance explained for “Satisfaction with Treatment” module**

| Components | Extraction sums of squared loading | | | Rotation sums of squared loading | | |
| --- | --- | --- | --- | --- | --- | --- |
|  | Eigenvalue | Variance contribution rate, % | Accumulated variance contribution rate, % | Eigenvalue | Variance contribution rate, % | Accumulated variance contribution rate, % |
| 1 | 3.56 | 39.55 | 39.55 | 3.56 | 39.54 | 39.54 |
| 2 | 1.85 | 20.51 | 60.07 | 1.85 | 20.53 | 60.07 |
| 3 | 0.96 | 10.67 | 70.74 |  |  |  |
| 4 | 0.74 | 8.19 | 78.93 |  |  |  |
| 5 | 0.55 | 6.15 | 85.08 |  |  |  |
| 6 | 0.48 | 5.36 | 90.44 |  |  |  |
| 7 | 0.39 | 4.32 | 94.76 |  |  |  |
| 8 | 0.30 | 3.30 | 98.06 |  |  |  |
| 9 | 0.18 | 1.94 | 100.00 |  |  |  |

**Supplementary table 4. Rotated factor matrix of the “Satisfaction with Treatment” module (promax method)**

| Items | Factors | | |
| --- | --- | --- | --- |
|  | 1 | 2 |  |
| E1 | **0.796** | 0.059 |  |
| E2 | **0.716** | 0.158 |  |
| E3 | **0.827** | -0.113 |  |
| E4 | **0.848** | 0.142 |  |
| E5 | **0.593** | -0.172 |  |
| E6 | **0.687** | -0.181 |  |
| E7 | 0.392 | **0.514** |  |
| E8 | -0.138 | **0.792** |  |
| E9 | -0.110 | **0.913** |  |

Note: values in boldface indicated the highest factor loading of the item.
